# Biochemical, Genotoxic and Histological Implications of Polypropylene Microplastics on Freshwater Fish *Oreochromis mossambicus*: An Aquatic Eco-Toxicological Assessment

**DOI:** 10.3390/toxics11030282

**Published:** 2023-03-19

**Authors:** Jeyaraj Jeyavani, Ashokkumar Sibiya, Thambusamy Stalin, Ganesan Vigneshkumar, Khalid A. Al-Ghanim, Mian Nadeem Riaz, Marimuthu Govindarajan, Baskaralingam Vaseeharan

**Affiliations:** 1 Biomaterials and Biotechnology in Animal Health Lab., Department of Animal Health and Management, Alagappa University, Karaikudi 630003, Tamil Nadu, India; 2 Department of Industrial Chemistry, Alagappa University, Karaikudi 630003, Tamil Nadu, India; 3 Department of Zoology, College of Science, King Saud University, Riyadh 11451, Saudi Arabia; 4 Texas A&M University, College Station, TX 77843, USA; 5 Unit of Mycology and Parasitology, Department of Zoology, Annamalai University, Annamalainagar 608002, Tamil Nadu, India; drgovind1979@gmail.com; 6 Unit of Natural Products and Nanotechnology, Department of Zoology, Government College for Women (Autonomous), Kumbakonam 612001, Tamil Nadu, India

**Keywords:** polypropylene microplastics, freshwater ecosystem, ever-growing threats, liver tissues, food, genotoxicity

## Abstract

In recent years, polypropylene microplastic has persisted in freshwater ecosystems and biota, forming ever-growing threats. This research aimed to prepare polypropylene microplastics and evaluate their toxicity to the filter feeder *Oreochromis mossambicus*. In this research, fish were given a dietary supplement of polypropylene microplastics at 100, 500, and 1000 mg/kg for acute (96 h) and sub-acute (14 days) durations to assess toxic effects on liver tissues. FTIR results revealed the presence of polypropylene microplastic in their digestion matter. The ingestion of microplastics in *O. mossambicus* led to fluctuations in homeostasis, an upsurge in reactive oxygen species (ROS) levels, an alteration in antioxidant parameters, including superoxide dismutase (SOD), catalase (CAT), glutathione-S–transferase (GST), and glutathione peroxidase (GPx); a promotion in the oxidation of lipid molecules; and a denaturation in the neurotransmitter enzyme acetylcholinesterase (AChE). Our data indicated that sustained exposure to microplastics (14 days) produced a more severe threat than acute exposure (96 h). In addition, higher apoptosis, DNA damage (genotoxicity), and histological changes were found in the liver tissues of the sub-acute (14 days) microplastics-treated groups. This research indicated that the constant ingestion of polypropylene microplastics is detrimental to freshwater environments and leads to ecological threats.

## 1. Introduction

Microplastics may pose a hazard to a variety of ecosystems, making them a potential contaminant. Due to their diminutive size, they are readily diffused and carried throughout all aquatic environments, including sediment, the surface, and other zones such as the littoral, limnetic, profundal, euphotic, and benthic [1,2,3], and even in aquatic creatures [4]. In general, biota accidentally ingests microplastics, via oral, and gill, which cause obstruction or irritation of the digestive system due to their accumulation. Accumulated microplastics were transported into different internal organs via circulatory body fluids. Which triggers the dysfunction or alteration in endocrine and metabolic pathways, leads to oxidative stress, cell necrosis, finally inducing cell apoptosis, and aids the organism’s death [5,6]. Microplastics and their impacts were reported in algae, zooplankton, fish, marine reptiles, sea birds, and aquatic mammals [7,8,9,10,11]. Among these, fish is a crucial predator and is considered one of the most protein-rich food sources for humans. Hence, they considered themselves ideal organisms to access the pollutants’ toxic effects, especially microplastics particles. Moreover, the intake of microplastics by marine and freshwater fishes has been widely reported [10,11,12,13,14,15]. De Sá et al. [4] revealed that 77% of microplastic toxicity research focused only on marine organisms, whereas 23% of studies focused on freshwater animals. Additionally, freshwater habitats are the most crucial transporters of microplastics from land into marine habitats [16]. Hence, the present research chose the edible freshwater fish *O. mossambicus* to investigate the effects of microplastic exposure for up to fourteen days.

Majorly three types of microplastics found in aquatic systems comprise polyethylene, polypropylene, and polystyrene, according to Li et al. [10] and Jeyavani et al. [6]. The vast majority of researchon the toxicity of microplastics in fish was concentrated on polystyrene [17,18,19,20], polyethylene [21,22,23], and polyvinyl chloride [24,25,26]. Conversely, there is limited information on the impact of polypropylene microplastic on aquatic creatures [27,28]. In recent years, there has been a rise in the utilization of polypropylene thermoplastics as a result of their adaptability, low weight, resistance to heat, and necessary toughness, as well as their use in the food packaging, automobile, and textile industries, as well as for medical purposes, composite technology, optoelectronics, and the production of toys, among other applications [29,30]. As a result of incompatible disposal procedures, polypropylene polymers survive in the environment, converting into micro-sized particles [6]. These microparticles are carried by the wind, rain, and anthropogenic activities into many ecosystems [31].

In this scenario, the current study aimed to test the hypothesis of a dietary supplement of polypropylene microplastics in palatable freshwater organisms *O. mossambicus* for a period of 96 h and 14 days to determine its effects on biochemical markers such as antioxidant parameters, including superoxide dismutase (SOD), catalase (CAT), glutathione-S-transferase (GST), glutathione peroxidation (GPx), reactive oxygen species level (ROS), and oxidation of lipids (Lipid peroxidation-LPO). The liver tissues were chosen as the target organ for testing toxicological effects. In addition, the cellular damage, genotoxicity, and apoptosis of liver tissues were identified as indicators of the toxicity of polypropylene microplastics.

## 2. Materials and Methods

### 2.1. Microplastic Synthesis and Characterization

Following our prior procedure of Jeyavani et al. [32,33], commercially available polypropylene film (bags) was transformed into microplastics, whose size ranges from 11.86–44.62 µm. FTIR spectroscopy evaluated both commercially available polypropylene film (bags) and powdered microplastics. 

### 2.2. Fish Husbandry

*O. mossambicus* (juvenile age group) was procured from a fish farm in Karaikudi, Tamil Nadu, India. The fish were acclimatized (for up to two weeks) in Fibreglass Reinforced Plastics (FRP) tank (capacity: 1000 L) and maintained in the laboratory (12:12 h of light/dark) [34]. During the acclimation and experimental phases, multiparameter water quality metres (HORIBA U-52, Japan) were used to measure water parameters such as temperature (29.41 °C), pH (7.0 ± 0.3), salinity (0.025 ± 0.05 ppt), dissolved oxygen (6.9 ± 0.4% mg O_2_ L1), total ammonia (0.09 ± 0.01 mg N-NH_4_ L1), conductivity (342.6 ± 16.2% s/cm), and alkalinity (42.7± 6.2% mg Caco_3_). Additionally, the fish were given commercial pellets once a day. Prior to beginning the experiment, the fish were famished for 24 h.

### 2.3. Microplastics Exposure Design

The following procedures were used by Espinosa et al. [35] to simulate dietary supplements with polypropylene microplastics. Polypropylene microplastics (mg) were combined with commercial artificial feeds (kg) of dry food at nominal test doses [36], such as 100 mg polypropylene microplastics /kg of dry food (low-Group 1), 500 mg polypropylene microplastics/kg of dry food (medium-Group 2), and 1000 mg polypropylene microplastics/kg of dry food (high—Group 3). One percent of cod liver oil (binding agents) was then added to this mixture and vigorously shaken to ensure uniform distribution of microplastics on the commercial pellet. In the control group, artificial pellets with 1% cod liver oil were made without microplastic. Then they were shadily dried. The dried feeds were kept in a glass container.

Four tanks (control, group 1, group 2, and group 3) were used for the experimental setting. (Triplicate—totally required 12 tanks). In each tank, fish (n = 10) were maintained in 100 L glass aquaria for three days after acclimatization periods (for static movements). Then, control and test groups were frequently fed with our prepared feed formulations for 14 days. The fish were closely monitored during the supplementation periods to ensure that they consumed all the provided feed. At the end of 96 h (acute) and 14 days (chronic), from each tank (both control and microplastic exposure groups) randomly, three fish were sampled to perform the toxicity analysis. Dietary supplemented polypropylene microplastics were taken by *O. mossambicus* and proved by FTIR analysis of egestion materials.

### 2.4. Preparation of Samples for Biochemical Assay

Three fish were taken out and sacrificed from each tank at the end of 96 h (acute) and 14 days (sub-acute). The liver and brain organs were dissected, kept in ice-cold phosphate buffer saline (pH 7.2), and grained using a mortar and pestle. The obtained suspension was centrifuged (3000 rpm for 15 min). Before centrifugation, some amount of suspension was taken out for comet assay. The remaining suspensions were centrifuged, and the supernatant was used for further biochemical enzyme analysis. The obtained pellet was also suspended in phosphate buffer saline. Finally, the obtained suspension and supernatant were kept at −20 °C until further investigation. 

#### 2.4.1. Biochemical Assay

The biochemical enzyme examination of reactive oxygen species (ROS), antioxidant parameters (SOD, CAT GST, GPx), and oxidative stress (LPO) were performed on stored supernatants of liver tissues [both control and microplastic treated groups (group 1, 2, and 3). In addition, it accessed reactive oxygen species (ROS) because liver tissue is used as a major detoxifying organ that reacts with their enzymes to protect the organisms from harmful pollutants. And brain tissue extract was utilized to measure acetylcholine esterase (AChE) activity. Historically, the amount of proteins in liver and brain tissues was determined and measured by the Bradford method [37]. All tests were conducted using microtiter plate readers with 96 wells. Each test was conducted in triplicate.

##### ROS

96 microtiter well plates were used to perform the fluorometric analysis to determine the formation of ROS in polypropylene microplastic exposure groups after 96 h and 14 days [38]. Here, dichlorofluorescein diacetate (DCF) is the substrate. Briefly, 20 µL of liver homogenate was added together with 100 µL of phosphate buffer saline and 8.3 µL of dichlorofluorescein diacetate (dissolved in DMSO). The sample were then kept at 30 min for 37 °C. Excitation (485 nm) and emission (530 nm) wavelengths were used to determine the fluorescence intensity, and the DCF/mg protein values were calculated.

##### Antioxidant Parameters

In accordance with Suzuki [39], the superoxide dismutase (SOD) enzyme activity in liver tissues was evaluated at 560 nm (U/mg protein) using xanthine oxidase for 96 h and 14 days in the control and microplastic exposure groups. Following Cohen et al.’s [40] procedure, the CAT enzyme activity was measured using hydrogen peroxide as a substrate at 240 nm and expressed as U/mg protein. In addition, Habig et al. [41] determined the GST activity (340 nm) of liver extract (96 h and 14 days) by 1-chloro-2, 4-dinitrobenzene as a substrate and expressing it as U/mg protein. The glutathione peroxidase (Gpx) activity was determined by Rotruck et al. [42], using liver tissue extract and Ellam’s reagent as substrate, whose activity was represented measured as U/mg protein and evaluated at 420 nm.

##### Oxidative Stress on Lipids

Malondialdehyde (MDA) formation was detected using thiobarbituric acid at 532 nm using liver tissue extract, and the results were expressed as mol MDA/mg protein using LPO activity [43].

##### Acetylcholinesterase (AChE) Activity

The level of neurotransmitter acetylcholinesterase (AChE) was investigated by Ellman et al. [44] using acetylcholine iodide as a substrate with brain tissue extracts from microplastic treated groups as well as control groups. As a substrate, acetylcholine iodide was used, and the resulting measurement was taken at 412 nm. The enzyme values were presented in the form of nmol/mg protein.

### 2.5. Histology of Liver

The liver tissues of both the control and microplastic treatment groups of *O. mossambicus* (14 days) were preserved in formalin at a concentration of 10%. In addition, each tissue was sectioned to a thickness of 4 µm after being immersed in paraffin wax. Afterward, they were stained by hematoxylin and eosin and viewed under a Nikon inverted microscope (Nikon Eclipse Ti × 100).

### 2.6. Detection of Live/Death Cells

Using acridine orange and ethidium bromide (AO/EtBr) staining of liver cell suspensions, determine the degree of cell damage generated by food supplementation with polypropylene microplastics in *O. mossambicus* [45]. After mixing together 25 µL of cell suspension with acridine orange and ethidium bromide staining aqueous solution in equal amounts, the mixture is allowed to incubate for 5 min. In order to study the cells, an inverted fluorescent microscope (Labomed TCM400 Trinocular) was used.

### 2.7. Comet Assay

The alkaline comet test, also known as single-cell gel electrophoresis, was performed by Sing et al. [46] and was subsequently improved by our earlier techniques [36], and these procedures were modified by our earlier studies to obtain a suspension of fish liver tissues (both microplastics exposure groups and the control group). Additionally, liver cell viability was determined using the trypan blue exclusion technique. Before anything else, make a 1.5% solution of high-melting agarose and pour it over a clean glass slide. After solidification, low-melting agarose was combined with 20 µL of liver tissue sample solutions on the same glass slide. The slide was then placed in lysis buffer solution (compare the 2.5 M NaCl, 100 mM EDTA, 10 mM Tris-HCl, 1% Triton X-100, 10% DMSO; pH 10) and stored in the refrigerator for the remainder of the night. The slides were then stored in an electrophoresis buffer chamber (10N NaOH, 200 mM EDTA; pH13) for ten minutes at 25 mV. The slides were then stored in a buffer designed to neutralize acids (0.4 M Tris-HCl, pH 7.5). The glass slide was allowed to dry naturally before being dyed with EtBr at a 10 µg/mL concentration. After the staining process was complete, the slide was stored in a dark location. After that, the slide was analyzed using a microscope equipped with an inverted fluorescent microscopic (Labomed TCM400 Trinocular) system (20×). The length of the olive tail (DNA damage) in the microplastics exposed groups was measured using Image J application.

### 2.8. Data Analysis

Using SPSS Package 16.0, the statistical analysis employed ANOVA (one-way variance) and Microsoft Excel aids to making the graphs. The mean values were examined using Duncan’s multiple range tests to determine if they were significant at *p <* 0.05. The findings were presented as the mean ± standard deviation of the three experimental triplicates.

## 3. Discussion

Here, we employ *O. mossambicus* [47], a commercially significant freshwater species, as a better model organism to assess the risks posed by pollutants such as pharmaceuticals, metals, nanoparticles, and microplastics. They are omnivorous filter feeders [48,49] and can effectively consume pollutant particles (did not differentiate between the pollutant and food sources). In addition, fish are the highest predators in the aquatic ecosystem’s food chain, transferring hazardous pollutants to various trophic organisms. As a result, they were regarded as the most suitable model species for assessing toxicity. The current research pointed to determine the harmful impacts of polypropylene microplastics on the edible freshwater fish *O. mossambicus*.

The four types of microplastics that are most frequently detected in aquatic habitats include polyethylene, polypropylene, polystyrene, and polyethylene terephthalate [6]. The majority of research focused on the harmful effects of polystyrene and polyethylene microplastics on fish [18,50,51,52]. A minimal study has been done on the impact of polypropylene microplastics on fish [28] and other invertebrates [27,33].

This work was created by combining commercial pellets with polypropylene microplastic and binding it with cod liver oil. Similarly, Zhu et al. [36] and Espinosa et al. [35] have shown that food supplements may be used to feed microplastics. In addition, the liver tissues of *O. mossambicus* were selected as the target organs to assess polypropylene microplastics’ toxicity. Since liver tissues in aquatic vertebrates are responsible for detoxification, when vertebrates are exposed to dangerous pollutants, their tissues will have strong stress reactions [53].

### 3.1. Physiochemical Characterization of Microplastics

**Figure 1:**
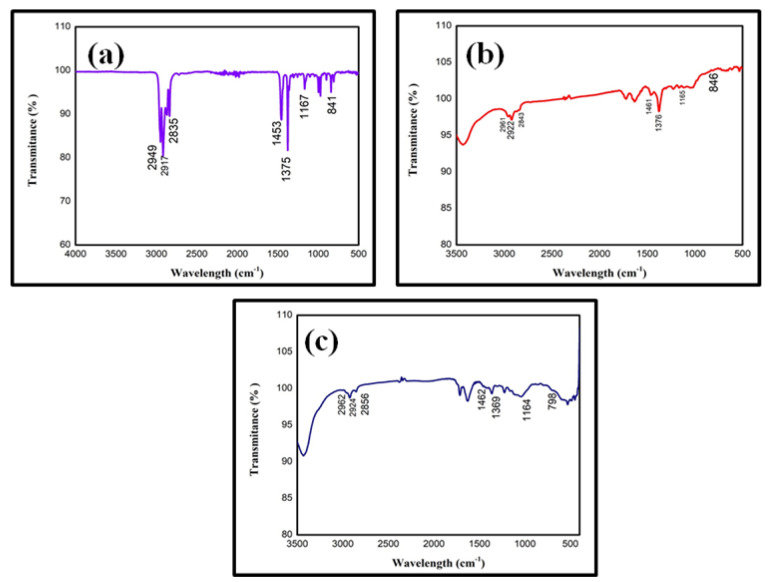
FTIR spectral graph of polypropylene. (**a**) Virgin polypropylene plastic film, (**b**) Polypropylene microplastics, (**c**) Polypropylene microplastics in fish’s egestion.

FTIR spectroscopy helps detect the chemical bonds in polypropylene plastics. The virgin polypropylene plastic film, powdered microplastics, and egestion material of *O. mossambicus* (Figure 1) showed similar peak values were observed in the FTIR spectrum at 2950–2860 cm^−1^ (CH_3_ stretching); 1453–1380 cm^−1^ (CH_3_ stretching); 1164–1165 cm^−1^ (C-H stretching); 798–846 cm^−1^ (C-H and CH_3_ stretching, C=C bending, −H-C-H bending). Similar to our findings, Tiwari et al. [54] and Sathish et al. [55] revealed the FTIR peak value of polypropylene polymers.

### 3.2. Reactive Oxygen Species

**Figure 2:**
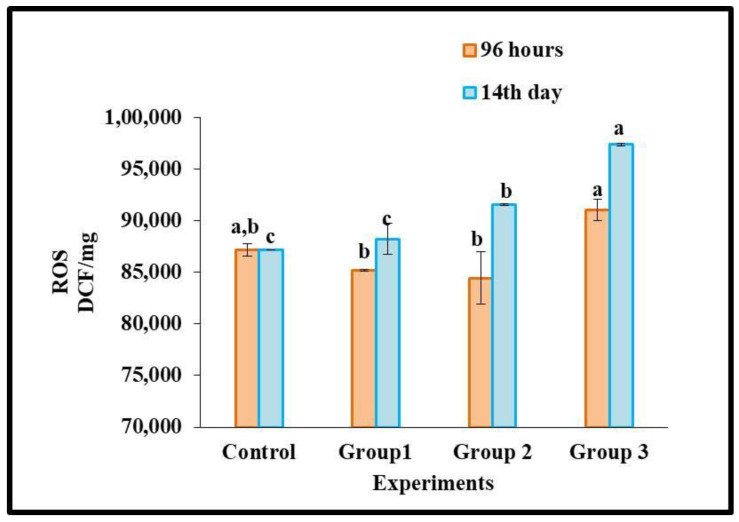
Generation of Reactive oxygen species (ROS) after 96 h and 14 days of dietary exposure to polypropylene microplastics [various concentrations of 100 mg/ kg (Group 1), 500 mg/kg (Group 2), 1000 mg/kg (Group 3)] in the liver tissues of *Oreochromis mossambicus* (Duncan’s multiple range tests significant value (*p* < 0.05). Statistically significant differences (*p* < 0.05) between experimental groups correspond to different alphabet letters.

When microplastic exposure to organisms can produce oxidative stress in the detoxification organ due to the formation of unpaired electrons in oxygen, the organisms rapidly increase ROS. Moreover, ROS levels are governed by mitochondria in the bodies of organisms. When ROS level increases, exposure to microplastics in organisms indicates damage to mitochondria [56]. This study reported polypropylene microplastics exposure and ROS levels in *O. mossambicus* fish’s liver tissues after 96 h and 14 days of exposure in Figure 2. The ROS level was decreased in the 96 h microplastic exposure groups (100 mg/kg and 500 mg/kg) and raised in the high-dose exposure group (1000 mg/kg) compared with the control group.

In addition, the ROS level in *O. mossambicus* rose continuously (*p*≤ 0.05) after being exposed to polypropylene microplastic in the food for 14 days (100, 500, and 1000 mg/kg). When there is an excess of ROS, the body’s natural redox equilibrium is disrupted. Hence, alterations in antioxidant status and oxidative stress are inevitable outcomes. In support of our results, ROS production was increased in goldfish larvae when exposed to polystyrene microplastic (1000 μg/L) for 7 days [57].

### 3.3. Antioxidant Biomarkers

In this study, dietary-supplemented polypropylene microplastic particles were consumed by *O. mossambicus* and entered the body via the gills and orally. Accumulated ingested plastic particles were in various body parts, such as the gastrointestinal tract, liver, tissues, gallbladder, etc. [57]. Free radicals are produced when microplastics accumulate in organisms, which is followed by a dramatic increase in ROS and an oscillation in the activity of antioxidant enzymes. [11].

**Figure 3:**
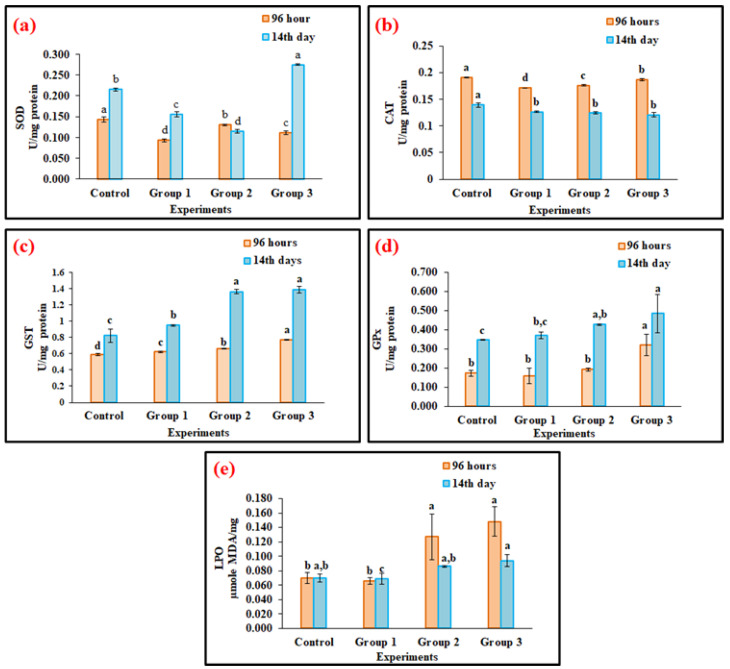
The antioxidant enzymes (**a**) Superoxide dismutase (SOD), (**b**) Catalase (CAT), (**c**) glutathione S-transferase (GST), (**d**) glutathione peroxidase (GPx) activities, and (**e**) oxidative stress effects on lipid peroxidation (LPO) in the liver tissues of freshwater filter-feeding organisms *Oreochromis mossambicus* after 96 h and 14 days dietary exposed of polypropylene microplastics at different concentrations (100 mg/kg (Group 1), 500 mg/kg (Group 2), 1000 mg/kg (Group3)). Statistically significant differences (*p* < 0.05) between experimental groups correspond to different alphabet letters. (Duncan’s multiple ranges test significant value *p* < 0.05).

In fish, superoxide dismutase (SOD) is the first line of defense against oxidative stress because it converts the free radicals (SO^−^) into hydrogen peroxide (H_2_O_2_). Likewise, in this study, *O. mossambicus* exposed to polypropylene microplastics (100, 500, and 1000 mg/kg) for 96 h (acute) and 14 days (subacute), liver antioxidant enzyme SOD level difference didn’t occur (Figure 3a) in the 96 h exposure groups compared with the control group. Moreover, in 14 days of microplastics exposure to groups 1 (100 mg/kg) and 2 (500 mg/kg), the SOD level decreased; it was more elevated in group 3 compared with the control group (*p* < 0.05) due to *O. mossambicus* protecting the body from oxidative stress. Similar outcomes were detected by Wen et al. [58,59] described that response to polystyrene microplastics in *Symphysodon aequifasciatus* enhanced the production of SOD level.

H_2_O_2_, a byproduct of SOD, is converted into water and oxygen by the second antioxidant enzyme, CAT. In this investigation, CAT activity was significantly decreased (*p* < 0.05) in 96 h and 14 days of polypropylene microplastics exposure in *O. mossambicus* groups compared with the control groups (Figure 3b) except in microplastic exposure group 3 (1000 mg/kg) of 96 h. Moreover, with the reduction of CAT enzyme activity, the fish produced a larger quantity of hydrogen peroxide and an imbalance in ROS formation in the liver cells. These results elucidate that polypropylene microplastic ingestion in *O. mossambicus* leads to impairment of the antioxidant defense system and oxidative damage. Similarly, Wang et al. [60] stated that 10 µm of polystyrene microplastics intake by *Oryzias melastigma* for up to 60 days led to impairment of the antioxidant defense enzyme CAT.

Glutathione-S-transferase (GST) is a crucial antioxidant enzyme concerned with phase 2 detoxification of cells from oxidative stress. GST, which is involved in the phase II detoxification process, prevents damage to cellular biological molecules by accelerating glutathione’s binding to foreign substances such as MPs [11]. In this investigation, GST activity in liver extracts of acute and sub-acute exposure to polypropylene microplastics in different groups (100, 500, and 1000 mg/kg) was significantly increased (*p* < 0.05) at 14 days and slightly raised (*p* < 0.05) in 96 h compared with the control groups (Figure 3c). The increased activity of GST levels is due to the protection of cells from oxidative damage from polypropylene microplastic exposure in *O. mossambicus*. Similarly, Huang et al. [61], Solomando et al. [62] and Romano et al. [26] showed comparable outcomes for microplastics exposure in freshwater fishes.

The decomposition of hydrogen peroxide is converted into fewer toxic hydroxyl molecules by Glutathione peroxidase (GPx) enzyme that helps protect the cells from oxidative damage. Similarly, the GPx activity also expressedly (*p* < 0.05) increased in microplastic exposure groups 3 (1000 mg/kg) in the acute period (Figure 3d), and other group 1 and group 2 (100 and 500 mg/kg) were similar to control at 96 h. The decrease in GPx level may be due to ROS accumulation of liver tissue in chronic polypropylene microplastic exposure groups [63]. Moreover, Yang et al. [56] reported that seven days of exposure to microplastics (1000 µg/L) down-regulated the GPx activity. Likewise, polystyrene microplastics (10 and 100 µg/L) exposed for up to 35 days in *Danio rerio* decreased the GPx activity [64], which may be due to the rise in ROS production from neutralizing hydrogen peroxide. However, in the chronic stage, the Gpx enzyme level was increased compared with the control groups (Figure 3d) due to the body’s protection from the induction of ROS level. Similarly, Huang et al. [61] and Solomando et al. [61], obtained comparable findings.

### 3.4. Oxidative Stress on Lipids

Malondialdehyde (MDA) is the lipid by-product of oxidative damage generation [64] and is a LPO biomarker. When fish are exposed to microplastics, it raises ROS levels. Furthermore, it causes inflammation and cell death. The formation of oxygen-free radicals triggered the LPO activity. In the current study, the MDA concentration significantly (*p* < 0.05) raised in both acute (96 h) and sub-acute (14 days) exposure to polypropylene microplastics (group 1–100 mg/kg, group 2–500 mg/kg, group 3–1000 mg/kg) treated groups due to oxidative damage or stress of lipids (Figure 3e). Similar to our findings, microplastic exposure dramatically increases LPO activity in *Dicentrarchus labrax* [65] and *O. niloticus* [66]. It increases the end product of (MDA level) LPO, resulting in tissue damage (cell membrane damage) due to oxidative stress.

### 3.5. AChE Activity

**Figure 4:**
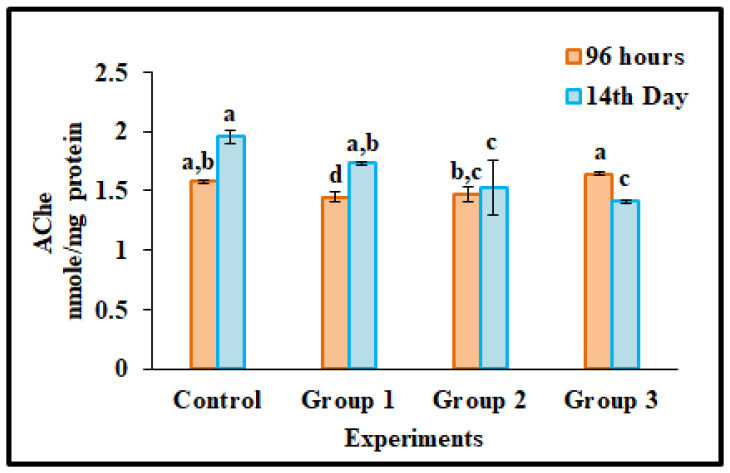
Neurotransmitter enzyme acetylcholinesterase activity (AChE) in brain tissues of *Oreochromis mossambicus* after 96 h and 14 days of dietary exposure to polypropylene microplastics at different concentrations (100 mg/kg (Group 1), 500 mg/kg (Group 2), 1000 mg/kg (Group 3)). Statistically significant differences (*p* < 0.05) between experimental groups correspond to different alphabet letters. (Duncan’s multiple ranges test significant value *p* < 0.05).

AChE delivered the neuromuscular chlorogenic destruction and was involved in the inactivation of acetylcholine, which helps in the transmission of cholinergic impulses in the neuromuscular junction of the cholinergic brain synapses [11]. Exposure to microplastics in fish prompted neurotoxicity that affects nerve-related enzymes. Generally, inhibition of the AChE level in the brain significantly alters the function of the nervous system [67]. Likewise, in the current study, AChE activity was accessed in the brain tissue of polypropylene microplastic-treated groups (100 mg/kg, 500 mg/kg, 1000 mg/kg) and untreated groups (96 h and 14 days). In 96 h of dietary microplastics exposure, group 3 (1000 mg/kg) gradually increased the AChE activity, but in group 1 (100 mg/kg) and group 2 (500 mg/kg), no differences were observed compared with the untreated groups (*p* < 0.05) (Figure 4). In contrast, in the 14 days of polypropylene microplastics exposure groups, AChE activity decreased progressively compared with the control group. Fourteen days of exposure to polystyrene microplastics in *O. niloticus* causes neurotoxicity [17] due to damaging lipid peroxide enzymes or acetylcholine accumulating in the synapses [65]. It leads to motor dysfunction, neurotransmission disorders, and behavioral abnormalities [11,68].

### 3.6. Histology

**Figure 5:**
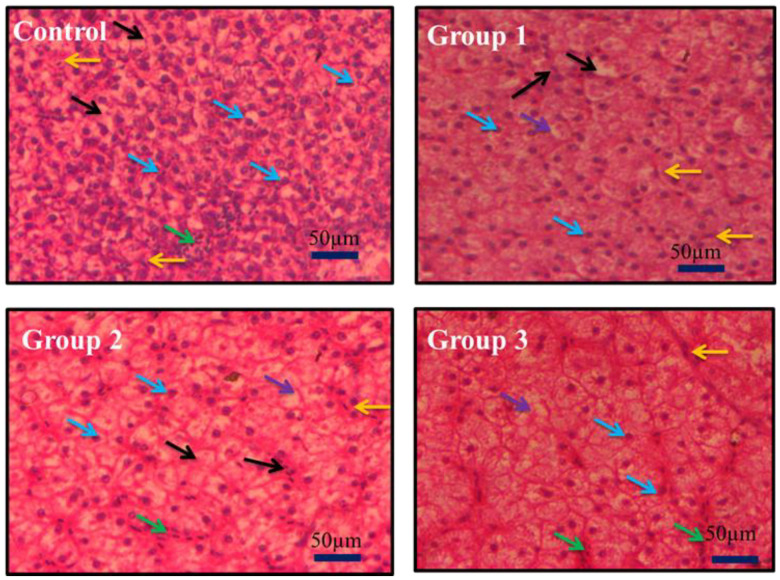
Representative histological liver sections [Phase contrast Nikon microscope magnification at 40×] of *Oreochromis mossambicus* exposed to polypropylene microplastics after 14 days at 100 mg/kg (Group 1), 500 mg/kg (Group 2), 1000 mg/kg (Group 3). Black arrow—Hepatocytes; Yellow arrow- Kupffer cells; Blue arrow—Infiltration of sinusoids with leukocytes; Green arrow—dilated sinusoids; Violet arrow—vacuole in hepatocytes. Compared with the control group, necrosis of hepatocytes will happen in the microplastics-treated group based on the concentration.

Polypropylene microplastic exposure (14 days) groups (Group 1—100 mg/kg; Group 2—500 mg/kg; Group 3—1000 mg/Kg) and the control group’s liver tissues of *O. mossambicus* were chosen for histological analysis to study the cellular damages. The histology of liver tissues is reported in Figure 5. From it, polypropylene microplastic exposure grouped organisms show a reduction in sinusoid infiltration with leukocytes, necrosis of hepatocytes, production of vacuoles in hepatocytes, and atrophy of dilated sinusoids. Based on the microplastic dietary doses, damage to liver cell components was observed. In addition, the distribution of hepatocytes also changed. The destruction of liver cells was directly proportional to the polypropylene microplastic exposure doses. In support of our results, polyvinyl chloride and polyethylene microplastics exposed to European sea bass for up to three weeks cause liver tissue damage [35]. Current study outcomes display that dietary consumption of polypropylene microplastics for up to 14 days in *O. mossambicus* generates ROS levels that lead to oxidative stress that causes damage to liver tissues.

### 3.7. Live/Dead Assay for Liver Tissues

**Figure 6:**
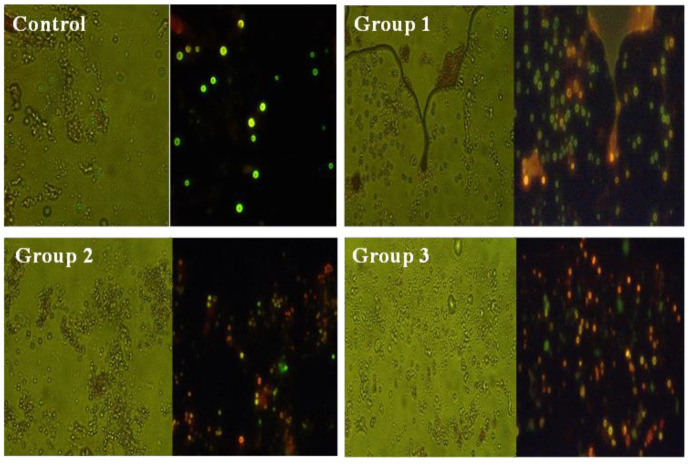
AO/EtBr fluorescent staining of *Oreochromis mossambicus* (liver tissues) treated with 14 days of polypropylene microplastics treated groups [100 mg/kg (Group 1), 500 mg/kg (Group 2), 1000 mg/kg (Group 3)] and control group showing healthy cells—green colour; unhealthy cells—orange and red colour.

AO/EtBr labeling helps distinguish between living and nonliving cells in tissue samples. The AO stain rapidly penetrates the cell membrane and reaches the nucleus, where it bonds with DNA to generate a green fluorescent color (indicating live cells); the EtBr stain only penetrates damaged cells and produces an orange fluorescent color. Early apoptotic cells are shown yellow with condensation of nuclear chromatin, and late apoptotic entire cells have red nuclei with complete structure. In this study, after AO/ EtBr staining, three types of cells were distinguished under the fluorescent microscope (Figure 6). Namely, green color (healthy cells), red color (unhealthy cells) and yellowish color (pre-unhealthy cells) were distinguished under fluorescent microscope staining with AO/EtBr staining. It is evident from Figure 6 that the control group only saw green (healthy) fluorescently stained cells. It denoted that the cells were live. Compared with the control group, up to 14 days of dietary supplementation with polypropylene microplastics in group 1 (100 mg/kg), almost all the cells were green in color, some of them yellowish color and few of them red; in group 2 (500 mg/kg), many of the liver cells are a yellowish color and red, and a few cells are red; in group 3, (1000 mg/kg) the most of liver cells are red. Few of the yellowish color cells were observed. This result showed that dietary ingestion of polypropylene microplastics in *O. mossambicus* induces oxidative damage to cells.

### 3.8. Comet Assay

**Figure 7:**
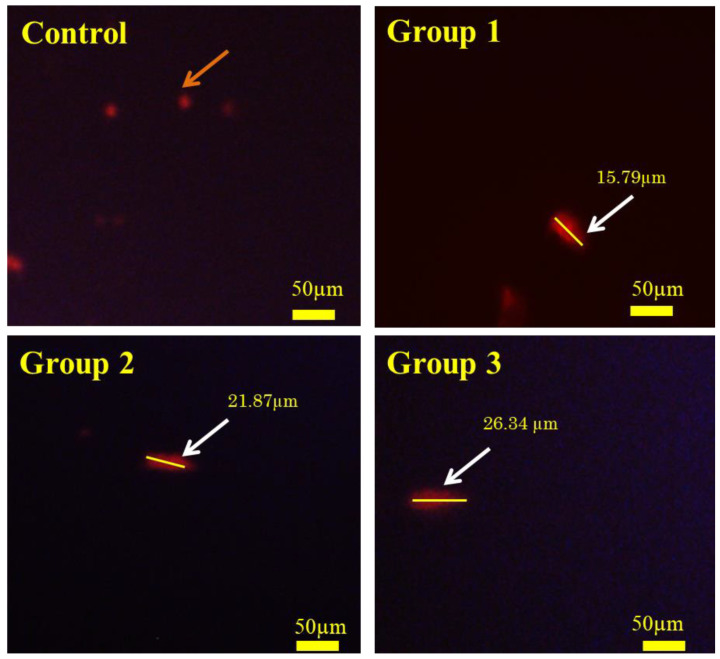
Fluorescent images of comet assay in *Oreochromis mossambicus* liver tissue of 14 days polypropylene microplastics treated groups [100 mg/kg (Group 1), 500 mg/kg (Group 2), 1000 mg/kg (Group 3)] showing the increasing degree of DNA damage (magnification: 20×) and the arrow indicates the DNA damages (Orange arrow-Oval-shaped; white arrow-comet-shaped) of liver cells.

Consumption of microplastics by aquatic organisms causes genotoxicity through ROS production in their bodies [69]. DNA damage (comet-shaped cells) was determined through the comet assay. The comet assay helps to identify the DNA damage in single and double standards using alkaline elution [34,70]. In our research, the ROS amount dramatically increased in 14 days of polypropylene microplastics exposure groups (Group 1—100 mg/kg; Group 2—500 mg/kg; Group 3—1000 mg/Kg) of *O. mossambicus*. From Figure 7, results showed that dietary ingestion of polypropylene microplastic increased the DNA damage directly proportional to microplastics exposure doses. In the control group, the DNA of the liver cells was condensed (visualized in a round shape). Nevertheless, in the microplastic exposure group, the DNA of the cells is stretched out and looks comet-shaped. The findings demonstrated that a dietary supplement of polypropylene microplastics for 14 days induces DNA damage in *O. mossambicus*. It may induce apoptosis or cell death in liver tissues. The length of the olive tail of microplastics exposure groups 1, 2, and 3—15.79 µm, 21.87 µm, and 26.34 µm, respectively. Likewise, ingesting polystyrene microplastics in freshwater fish *Danio rerio* and *Perca fluviatilis* causes DNA damage in the liver and gill tissue [71].

### 3.9. Proposed Toxicological Mechanisms of Dietary Exposure (14 Days) of Polypropylene Microplastics in O. mossambicus

In this study, prolonged exposure (14 days) to microplastics enters into the bodies of the organisms, which could disrupt redox homeostasis. The organisms that were exposed for 96 h protected themselves from the disruption of redox-homeostasis with the help of an antioxidant system [72] and restored the balance of homeostasis. However, after 14 days of stress, organisms could no longer return to homeostasis. This disruption causes swift generation of ROS, i.e., the production of unpaired electrons in oxygen (Figure 2), depending on the polypropylene dietary exposure level in *O. mossambicus.* Raised the ROS level, which initially affects the physiological oxidative pathways [73]. This unpaired electron participates in the oxidative antioxidant pathway for neutralizing redox homeostasis. To neutralize the excess ROS, the antioxidant enzyme was adequately increased. This study gradually increases antioxidant enzymes such as SOD, GST, and GPx activities (Figure 3a–d). Briefly, the formed free radicals react with the primary defense enzyme Superoxide dismutase (SOD) which converts them into hydrogen peroxide (H_2_O_2_) (Figure 3a). The higher production of H_2_O_2_ reduced the CAT enzyme in this study (Figure 3b). Furthermore, it was noted that the increased GST and GPx activities protect the liver cells from oxidative damage (Figure 3c,d). Our findings are supported by studies showing that exposing Cyprinus carpio to herbicides, hydrocarbons, and metals causes oxidative stress and tissue damage [74,75,76]. Further elevation of ROS, downregulation of SOD, CAT, and GPx, oxidative stress, and skin injury in mouse skin tissues were seen after simultaneous exposure to microplastics and plasticizers [77].

**Figure 8:**
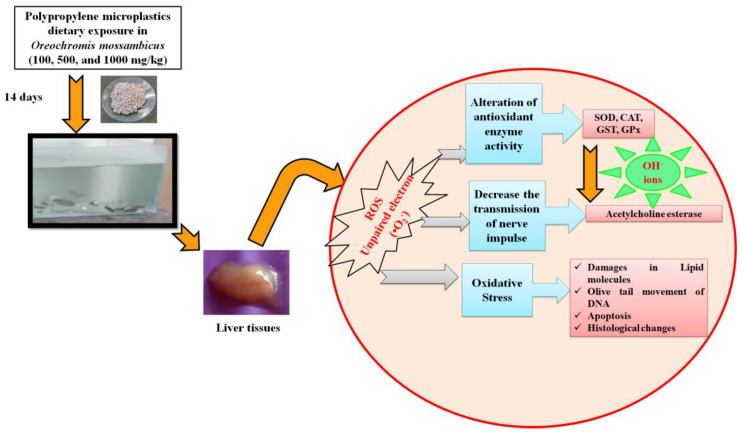
Interaction of polypropylene microplastics and antioxidant system of *O. mossambicus* toxicological pathway and their proposed mechanisms based on the outcome of this study.

Finally, the formation of hydroxyl ions affects biomolecules in the cells [78]. Likewise, in the current study, the oxidation of lipid biomolecules led to the production of MDA (lipid by-product) (Figure 3e) in liver tissues. This showed the lipid cells were damaged in liver tissues [71]. Furthermore, it causes the apoptosis of liver cells (Figure 6), and oxidation of DNA leads to the olive tail movement (Figure 7). Finally, tissue damage was also confirmed by the histology of the results (Figure 5). These histology results elucidate the reduction in sinusoid infiltration with leukocytes, necrosis of hepatocytes, production of vacuoles in hepatocytes, and atrophy of dilated sinusoids. Overall, this mechanism elucidates the disruption of redox homeostasis by increasing the unpaired electron in oxygen, which reacts with the antioxidant enzyme (maintains normal homeostasis). Overproduction of this free radical aids in the generation of hydroxyl ions, which damage proteins, lipids, neurotransmitter enzymes, and DNA, and finally resulting in the death of the organisms (Figure 8). The current results and their mechanistic pathway strongly support the proposed initial hypothesis and further confirm that prolonged exposure to pollutants causes severe effects compared with acute exposure.

## 4. Conclusions

Redox metabolism was shown to be altered in *O. mossambicus* when polypropylene microplastics were supplemented into the diet. Consequently, generating the ROS level leads to fluctuations in antioxidant biomarkers (SOD, CAT, GST, and GPx) and promotes the oxidation of lipid biomolecules in liver tissues. The results indicated that prolonged polypropylene microplastic consumption (14 days) by *O. mossambicus* is more dangerous. Furthermore, 14 days of exposure to microplastic-treated organisms increased cell death and DNA damage in liver tissues. Moreover, histological results showed that morphological changes included a reduction of infiltration of sinusoids with leukocytes, necrosis and production of vacuoles in hepatocytes, and atrophy of dilated sinusoids in the liver of *O. mossambicus*. Overall, the outcome of this study elucidated that prolonged ingestion of polypropylene microplastics by aquatic organisms collapses the complete food web. Future research will focus on better remedial practices for destroying microplastics in the aquatic ecosystem.

## Data Availability

Not applicable.
